# Age and gender differences in seven tests of functional mobility

**DOI:** 10.1186/1743-0003-6-31

**Published:** 2009-07-30

**Authors:** Annie A Butler, Jasmine C Menant, Anne C Tiedemann, Stephen R Lord

**Affiliations:** 1 Prince of Wales Medical Research Institute, University of New South Wales, Barker St, Randwick, Sydney, NSW, 2031, Australia

## Abstract

**Background:**

The objective of this study was to examine age and gender differences in seven tests of functional mobility.

**Methods:**

The study included 50 young participants aged 20 to 39 years, and 684 older participants aged 75 to 98 years. Functional mobility measures included the coordinated stability test, the near tandem balance test, the six metre walk test, the sit to stand test with five repetitions, the alternate step test and the stair ascent and descent tests.

**Results:**

Older participants performed significantly worse than the younger participants in all of the functional mobility tests (p < 0.001), with the older women performing worse than the older men in all of the tests (p < 0.05). Significant correlations were found within the older group among all the functional mobility tests scores (r = 0.24–0.87, p < 0.001), and between functional mobility performance and age (r = 0.14–0.35, p < 0.001). People with arthritis and stroke performed worse than people without these conditions in these tests.

**Conclusion:**

This study provides a normative database for performance of young and older community-dwelling people in a battery of validated and reliable functional mobility tests. The results confirm age-related differences in functional mobility between young and older adults.

## Background

Mobility tests are commonly used to assess function and frailty in older populations. Many of these tests are also used with younger adults as measures of physical fitness and general health; however there are little data available on the age-related changes in the performance of these tests.

Several studies have shown that there is a decline in the ability to perform balance-related tests as age increases [1-3] with a significant decline commencing at approximately 40 years of age [4,5]. Similarly, gait speed slows with age [6,7] and the ageing process contributes to declines in stair negotiation ability [8] and lower limb strength [9]. These age-related changes in the performance of functional mobility measures and physiological domains are also associated with an increased risk of falls, ongoing disability and admission into residential aged care [10].

The development of age stratified normative data for these commonly used functional mobility tests could assist in the targeting of interventions for people who exhibit a decline in their functional status at an early stage, prior to the occurrence of falls and the onset of disability. Therefore, the aim of this study was to provide reference data and examine age and gender differences in seven functional mobility tests. The second aim was to identify how much common age related diseases, i.e. arthritis and stroke, further impaired performance in these tests.

## Methods

### Participants

Fifty young participants (23 men) aged 20–39 years (mean: 28.4 ± 4.7 years) and 684 older people (238 men) aged 75 years and over (mean: 80.1 ± 4.4 years) performed seven tests of functional mobility. The young participants were a convenience sample of healthy staff members of the Prince of Wales Medical Research Institute. The older participants were randomly selected from the membership database of a health insurance company as part of a falls prevention randomised controlled trial conducted between 1999 and 2002 [11]. Exclusion criteria included minimal English, blindness, Parkinson's disease or a Short Portable Mental Status Questionnaire score <7 [12]. All participants were living independently. The mobility tests were carried out at an acute hospital and transport was provided for people with mobility limitations. Table 1 shows the prevalence of medical conditions, medication use and participation in physical activity of the older participants.

**Table 1:** Prevalence of major medical conditions, medication use and participation in physical activity in the older sample

**Measure score range**	**N or (Mean)**	**% or (SD)**
*Health Status*		

Arthritis	283	41.8

Diabetes	46	6.7

Incontinence	103	15.1

Depression	70	10.2

Stroke	48	7.0

Dizziness	30	4.4

SF-12 Physical Component Summary Score	(48.24)	(8.95)

SF-12 Mental Component Summary Score	(55.57)	(6.71)

*Medications*		

Psychoactive medications	105	15.4

Cardiovascular medications	477	69.7

Musculoskeletal medications	161	23.5

≥ 4 medications	377	55.1

		

Planned walk at least once/week	378	55.3

Use of walking aid	115	16.8

		

≥ 1 fall in the past year	294	43.0

Moderate or marked fear of falling	189	27.9

Thirty older participants undertook the tests a second time two weeks after their initial assessment to determine the test-retest reliability of the tests. The University of New South Wales Ethics Committee approved the study and informed consent was obtained from participants prior to their participation.

### Functional mobility tests

The seven tests were administered in a single session. Timed tests were measured with a stopwatch with an accuracy of 0.01s.

#### Coordinated stability

**Figure 1:**
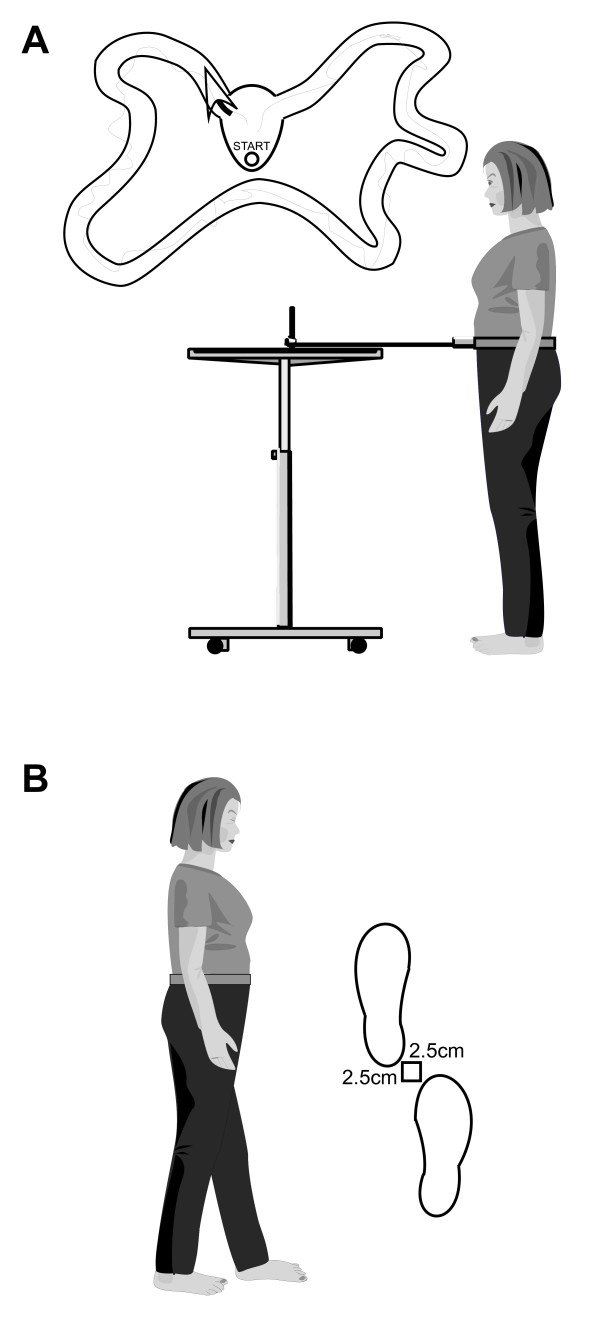
**Tests of (A) Coordinated stability and (B) Near tandem balance**.

The coordinated stability task measured participants' ability to adjust balance in a steady and coordinated way while placing them near or at the limits of their base of support (Figure 1A) [13]. This test used the Lord swaymeter – a simple device comprising a 40 cm rod which was attached to participants at waist level by a firm belt [14]. The participant was asked to adjust balance by bending or rotating the body without moving the feet (i.e. move the centre of mass), so that a pen mounted vertically at the end of the rod followed and remained within a convoluted track which was marked on a piece of paper attached to the top of an adjustable height table. To complete the test without errors, participants had to remain within the track, which was 1.5 cm wide, and be capable of adjusting the position of the pen 29 cm laterally and 18 cm in the anterior-posterior plane. A total error score was calculated by summing the number of occasions that the pen on the swaymeter failed to stay within the path. Where participants failed to negotiate an outside corner (because they could not adjust their centre of mass sufficiently), five additional error points were accrued. Participants completed a practice trial before completing the test.

#### Near tandem balance

In this test, participants were asked to stand in a near tandem position with their bare feet separated laterally by 2.5 cm with the heel of the front foot 2.5 cm anterior to the great toe of the back foot (Figure 1B). Participants chose which foot to place in the forward position for the test and they were required to stand in this position for 30s with eyes closed. The time that participants were able to stand in this position before a step was taken or the eyes were opened was the score. If a score of 5s or less was obtained, a second trial was allowed and the better result was used as the test score.

#### Walking speed – six metre walk

Participants were asked to walk along a straight, flat, well-lit corridor at their "normal walking speed". Two markers were used to indicate the start and end of the 6 m path and a 2 m approach was allowed before reaching the start marker so that participants were walking at their normal pace within the timed path. The participants were also instructed to continue walking past the end of the 6 m path for a further 2 m, to ensure that the walking pace was kept consistent throughout the task. Walking speed (m/s) was used as the test measure.

#### Sit to stand

In this test, participants were asked to rise from a standard height (43 cm) chair without armrests, five times as fast as possible with their arms folded. Participants undertook the test barefoot. The time from the initial seated position to the final seated position after completing five stands was the test measure.

#### Alternate step

The alternate step test is a modified version of the Berg stool stepping task [15]. It involves weight shifting and provides a measure of lateral stability. This test involved alternatively placing the whole left and right foot (shoes removed) as fast as possible onto a step that was 19 cm high and 40 cm deep. The time taken to complete eight steps, alternating between left and right foot comprised the test measure.

#### Stair ascent and descent

In this study the test stairs were indoors, had a handrail, were covered with linoleum and well lit. The participants started the stair ascent test at the bottom of eight steps (15 cm high, 27.5 cm deep). Participants were instructed to complete the task as fast as possible and could use the handrail if preferred and a walking aid if they normally used one. Timing commenced for the stair ascent test when the subject raised their foot off the ground to climb the first step and stopped when both feet were placed on the eighth step (which was a landing). After a brief rest, participants were asked to descend the stairs. Timing was started when they raised their foot off the ground for the first step and stopped when they completed the last step. Times taken to complete the ascent and descent tests were recorded and converted to the number of steps taken per second.

### Statistical analysis

Test-retest reliability for the test measures was assessed with intra-class correlation (ICC_3,1_) tests. As not all test scores were normally distributed (particularly in the young participants), non-parametric statistics were used in all between-group comparisons. The relationships among the mobility tests were examined with Spearman correlations. Mann Whitney-U tests were used to assess differences in mobility task performance between young and older participants, between older participants with and without stroke and, with and without arthritis, as well as to assess gender differences within the young and older groups.

## Results

### Test-retest reliability

According to the criteria of Shrout and Fleiss [16], the ICC_3,1 _values determined from the older sample indicated excellent reliability for the sit to stand test (0.89, 95% CI = 0.79, 0.95), the coordinated stability test (0.83, 95% CI = 0.70, 0.91), the alternate step test (0.78, 95% CI = 0.59, 0.89), the stair ascent test (0.84, 95% CI = 0.69, 0.92) and the stair descent test (0.86, 95% CI = 0.74, 0.93). The six metre walk test and the near tandem balance test displayed good and fair reliability (0.74, 95% CI = 0.52, 0.87 and 0.54, 95% CI = 0.23, 0.75 respectively).

### Age and gender comparisons

Table 2 shows the median scores and interquartile ranges (IQR) for the young men and women and the older men and women when categorised into four age groups (75–79, 80–84, 85–89, 90+ years). The mean ages of the older men and women were very similar (80.0 ± 4.6 vs. 80.2 ± 4.4 years, p = 0.67). The older participants (as a group) performed significantly worse in all seven tests than their younger counterparts (p < 0.001). There were no differences in the test performances of young women and young men, however, older women performed worse than older men in all of the tests (p < 0.05).

**Table 2:** Median (IQR) functional mobility test scores for men and women in each age group

**Test (measure)**	**Age group** (years)	**Men** Median (IQR)	**Women** Median (IQR)	**Total** Median (IQR)
Coordinated stability (errors)	20–39	0.0 (0.0–0.0)	0.0 (0.0–0.0)	0.0 (0.0–0.0)

	75–79	2.0 (0.0–7.0)	5.0 (2.0–10.0)	4.0 (1.0–9.5)

	80–84	4.0 (0.5–8.0)	10.0 (4.0–17.0)	8.0 (3.0–15.5)

	85–89	8.0 (4.0–14.0)	12.5 (3.75–19.0)	11.0 (4.0–18.0)

	90+	15.0 (6.0–23.0)	16.0 (6.5–20.5)	15.5 (6.2–20.7)

	**Total (75+)**	**4.0 (0.0–10.0)**	**7.0 (3.0–15.0)**	**6.0 (2.0–13.0)**

Near tandem balance (s)	20–39	30.0 (30.0–30.0)	30.0 (30.0–30.0)	30.0 (30.0–30.0)

	75–79	15.8 (4.2–30)	9.2 (4.7–30)	10.8 (4.3–30.0)

	80–84	17.4 (4.8–30.0)	7.2 (2.8–29.2)	8.3 (3.0–30.0)

	85–89	5.0 (0.0–23.3)	3.9 (1.2–8.4)	3.9 (0.5–11.6)

	90+	0.0 (0.0–3.3)	0.0 (0.0–2.4)	0.0 (0.0–2.5)

	**Total (75+)**	**14.7 (3.4–30.0)**	**7.2 (3.0–29.6)**	**8.2 (3.1–30.0)**

Walking speed (m/s)	20–39	1.5 (1.2–1.6)	1.4 (1.3–1.6)	1.4 (1.3–1.6)

	75–79	1.1 (0.9–1.3)	1.1 (0.9–1.2)	1.1 (0.9–1.2)

	80–84	1.1 (0.9–1.2)	1.0 (0.9–1.4)	1.0 (0.9–1.2)

	85–89	1.1 (0.8–1.2)	0.8 (0.7–1.0)	0.9 (0.7–1.1)

	90+	0.9 (0.6–0.9)	0.8 (0.6–0.9)	0.8 (0.6–0.9)

	**Total (75+)**	**1.1 (0.9–1.2)**	**1.0 (0.9–1.1)**	**1.0 (0.9–1.2)**

Sit to stand (s)	20–39	7.9 (6.9–9.4)	8.0 (6.4–9.0)	7.9 (6.6–9.0)

	75–79	10.3 (9.0–12.9)	11.5 (9.3–13.6)	11.2 (9.1–13.4)

	80–84	11.5 (9.4–14.5)	12.0 (10.0–15.0)	11.9 (9.7–14.7)

	85–89	11.7 (9.8–14.7)	12.1 (10.2–15.0)	12.0 (10.1–14.9)

	90+	14.5 (9.7–30.0)	14.6 (10.7–15.2)	14.5 (10.5–20.6)

	**Total (75+)**	**10.9 (9.2–14.1)**	**11.9 (9.7–14.3)**	**11.6 (9.5–14.2)**

Alternate step (s)	20–39	6.9 (6.2–7.7)	6.8 (6.3–7.3)	6.8 (6.3–7.3)

	75–79	8.6 (7.5–10.6)	9.5 (7.8–10.9)	9.2 (7.7–10.9)

	80–84	9.3 (7.7–12.0)	10.7 (9.0–12.9)	10.2 (8.6–12.5)

	85–89	10.0 (8.5–13.2)	11.2 (9.1–16.4)	10.7 (8.8–15.4)

	90+	13.2 (9.5–18.8)	14.5 (12.2–20.8)	13.9 (11.7–20.2)

	**Total (75+)**	**9.1 (7.8–11.8)**	**10.1 (8.3–12.3)**	**9.7 (8.0–12.2)**

Stair ascent (steps/s)	20–39	2.0 (1.9–3.2)	2.6 (2.3–2.8)	2.5 (2.0–2.9)

	75–79	1.9 (1.6–2.2)	1.6 (1.4–2.0)	1.7 (1.4–2.0)

	80–84	1.8 (1.5–2.2)	1.4 (1.2–1.7)	1.5 (1.2–1.8)

	85–89	1.6 (1.4–1.8)	1.2 (0.8–1.5)	1.4 (1.0–1.6)

	90+	1.2 (0.9–1.5)	1.1 (0.5–1.6)	1.2 (0.9–1.6)

	**Total (75+)**	**1.8 (1.5–2.1)**	**1.5 (1.2–1.8)**	**1.6 (1.3–1.9)**

Stair descent (steps/s)	20–39	2.5 (2.2–3.9)	3.0 (2.7–3.4)	2.9 (2.3–3.4)

	75–79	2.0 (1.7–2.4)	1.6 (1.3–2.0)	1.8 (1.4–2.1)

	80–84	1.8 (1.4–2.2)	1.3 (0.9–1.7)	1.5 (1.1–1.9)

	85–89	1.7 (1.3–2.0)	1.2 (0.6–1.4)	1.3 (0.8–1.7)

	90+	1.0 (0.9–1.4)	0.9 (0.4–1.3)	1.0 (0.7–1.3)

	**Total (75+)**	**1.9 (1.4–2.3)**	**1.4 (1.1–1.8)**	**1.6 (1.2–2.0)**

Within the older group, performances in all of the mobility tests were significantly correlated (r = 0.24–0.87, p < 0.001), and all were weakly but significantly associated with age (r = 0.14–0.35, p < 0.001). In the young group fewer tests were significantly associated with each other (Table 3).

**Table 3:** Correlation coefficients (ρ) among the functional mobility tests.

	Coordinated stability	Near tandem balance	Walking speed	Sit to stand	Alternate step	Stair ascent	Stair descent	Age
Coordinated stability		-0.38***	-0.45***	0.32***	0.46***	-0.50***	-0.50***	0.30***

Near tandem balance	**0.11**		0.38***	-0.24***	-0.28***	0.31***	0.35***	-0.26***

Walking speed	**0.10**	**-0.01**		-0.49***	-0.57***	0.68***	0.65***	-0.30***

Sit to stand	**0.17**	**0.10**	**0.01**		0.63***	-0.54***	-0.51***	0.14***

Alternate step	**0.15**	**-0.09**	**-0.20**	0.49***		-0.64***	-0.64***	0.27***

Stair ascent	**-0.10**	**0.21**	0.39*	**-0.15**	-0.40**		0.87***	-0.33***

Stair descent	**0.09**	**0.22**	0.45**	**-0.14**	-0.35*	0.84***		-0.35***

Age	**-0.09**	-0.26*	**0.18**	**-0.17**	-0.25*	**0.09**	**0.03**	

The upper half represents the correlation coefficients for the older group. The bold lower half of the table represents correlation coefficients for the young group (* p < 0.05, ** p < 0.005, *** p < 0.001).

Two tests showed marked age differences. In the test of near tandem balance, all young participants were able to attempt the test and 94% completed the 30 second test period. In contrast, 11% of older participants were unable to attempt the test, and only 29% successfully completed it. In the test of coordinated stability, 84% of the young group recorded no errors, compared with just 15% of the older group.

During the stair ascent and descent tests, 45% and 52% (respectively) of older people held the handrail for assistance whereas only one young participant used the handrail in the test of stair descent.

**Figure 2:**
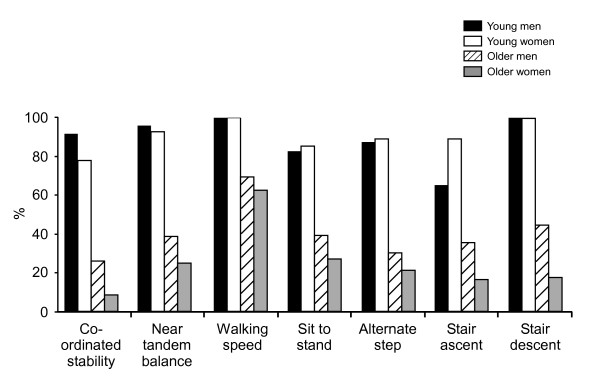
**Percentage of participants who performed each test adequately**. Reasonable performance levels for each test defined as: coordinated stability = 0 errors; near tandem balance = full 30s stand; walking speed ≥ 1 m/s; sit to stand ≤ 10s; alternate step ≤ 8s; stair ascent and descent ≥ 2 steps/s.

Figure 2 shows the percentage of young and older participants who could undertake each test within a time period or error level that indicated "reasonable" performance. This complementary reporting of the data also shows the large differences in test performances between the young and older groups.

### Medical conditions within the older group

Table 4 shows the median scores and interquartile ranges (IQR) for the older participants with and without stroke, as well as with and without arthritis. The older participants who had suffered from a stroke in the past had more than double the number of errors in the coordinated stability test than those who had not had a stroke. They also walked significantly slower and took longer time to complete the alternate step test and the stair ascent. The participants with arthritis performed significantly worse than those without arthritis in all the functional ability tests except for the near tandem balance test.

**Table 4:** Median (IQR) functional mobility test scores for participants with and without stroke and with and without arthritis (*p < 0.05, **p < 0.005, ***p < 0.001)

**Test (measure)**	**No stroke** **(n = 636)**	**Stroke** **(n = 48)**	**No arthritis** **(n = 401)**	**Arthritis** **(n = 283)**
Coordinated stability (errors)	5.5 (1.1–12.5)	11.5 (3.9–20.5)**	5.0 (1.0–12.1)	7.4 (2.9–14.6)***

Near tandem balance (s)	8.5 (3.1–30.0)	7.0 (2.0–30.0)	7.9 (3.1–30.0)	8.6 (3.0–30.0)

Walking speed (m/s)	1.1 (0.9–1.2)	1.0 (0.9–1.1)*	1.1 (1.0–1.3)	1.0 (0.9–1.2)***

Sit to stand (s)	11.5 (9.5–14.2)	12.1 (10.6–14.7)	11.0 (9.2–13.3)	12.5 (10.3–15.9)***

Alternate step (s)	9.7 (8.0–12.2)	10.6 (9.6–13.0)*	9.4 (7.8–11.4)	10.7 (8.4–13.4)***

Stair ascent (steps/s)	1.7 (1.3–2.0)	1.5 (1.3–1.7)*	1.8 (1.4–2.0)	1.5 (1.2–1.8)***

Stair descent (steps/s)	1.6 (1.2–2.0)	1.5 (1.1–1.8)	1.8 (1.4–2.1)	1.4 (1.0–1.9)***

## Discussion

When investigating age-related effects on functional mobility, a critical controversy arises relating to fundamental differences in the definition of the term "normal ageing". On the one hand, normal older people can be defined as only those free from all medical conditions, whilst on the other end, all older people, with no exclusion criteria and hence representative of the general population, can be considered normal. While both perspectives on selection criteria are valid, they lead to differing results, depending on whether pathological conditions are considered as a normal concomitant of the ageing process. The older sample on whom the data analysis was conducted was representative of the community-living older population and thus presented with a range of pathologies.

The study findings revealed significant age-related differences in all seven functional mobility tests examined. These findings confirm those of previous studies and indicate that when compared with young people, older people exhibit poorer leaning balance [1,2], more difficulty maintaining balance while standing with a reduced base of support [17], slower comfortable walking speed [6,7,10], reduced ability to quickly rise from a chair [10], and slower stair ascent and descent speed [18]. These age-related differences in functional mobility have been attributed to impaired sensorimotor function [19,20], in particular reduced lower extremity strength and power [19-22], but also to balance deficits [19,20], increased fear of falling [20,23] and reduced aerobic capacity [24].

Significant correlations among all the functional mobility tests in the older group indicate that older adults who performed poorly in one test were likely to perform poorly in all the other tests. This suggests that to a large extent these tests assess a common underlying "mobility" construct [25], rather than distinct functional abilities.

The finding that the older women performed worse than the older men in all the functional mobility tasks is in agreement with previous studies that have investigated lower-extremity functional performance [10], stair negotiation [20], rapid turns and stops [26], and is attributed to older women being less able to generate rapid lower limb muscle torques [20,26].

The tests differed considerably with regard to differences in performances between the young and older groups. The six metre walk test showed the smallest age difference and this is likely due to the test instruction requiring walking at normal rather than maximal pace, and the fact that this test is familiar and of low threat with respect to falling. In contrast, the stair descent test, which required participants to undertake the task as quickly as possible, is likely to have induced the greatest concern about falling and this was evident in different strategies adopted by the young and older participants. Only one young participant held the handrail while negotiating the stairs and many "ran" rather than walked down the stairs, while approximately half of the older people held the handrail and none adopted a running strategy. As 28% of the older sample reported moderate or marked fear of falling, this factor, in addition to sensorimotor function impairments, may have contributed to the large difference in stair descent speed between the young and older groups.

The greatest age-related differences in test performance were found in the coordinated stability and near tandem balance tests. These tests were completed without error by most young participants, but proved to be much more difficult for the older participants. This suggests that the ability to control and adjust standing balance may undergo greater age-related changes than transfer and walking tasks. However, it is also possible that the larger age effects may be partly due to familiarisation factors in that the coordinated stability and near tandem balance tests are less similar to everyday tasks than tests such as the sit to stand and stair negotiation which are integral elements of activities of daily living.

Normative data regarding functional mobility performance in older people suffering from two common medical conditions in our sample, stroke and arthritis, were also provided. As suggested in previous studies, sensory and motor control impairments likely contributed to reduced functional abilities in stroke survivors [27] and arthritis sufferers [28]. Surprisingly though, the difference in functional tests performance was not as large between stroke sufferers and non-stroke sufferers as it was between arthritis sufferers and non-arthritis sufferers. We did not assess the extent of damage and subsequent recovery from the stroke; it is likely that some of the older participants had functionally recovered from their stroke event which would explain the great variance in the scores. In contrast, the presence of arthritis would have been affecting the participants' mobility and balance on a daily basis.

## Conclusion

In conclusion, this study provides normative data for performance of young and older community-dwelling people in a battery of validated and reliable functional mobility tests. Significant age-related differences in performance were found in tests of coordinated stability, near tandem balance, six metre walk, alternate step, five-repetition sit to stand, and stair negotiation, with older women performing worse than older men in all tests.

## Competing interests

The authors declare that they have no competing interests.

## Authors' contributions

SL and AT conceived the study, participated in its design and coordination and tested the old participants. AB and JM carried out the testing of the young participants. AB performed the statistical analysis. All authors helped to draft the manuscript, read and approved the final manuscript.
